# Real-World Treatment Selection Factors and 7-Year Clinical Outcomes between Percutaneous Coronary Intervention and Coronary Artery Bypass Graft Surgery in Left Main Disease

**DOI:** 10.3390/jcm11030503

**Published:** 2022-01-19

**Authors:** Albert Youngwoo Jang, Minsu Kim, Joonpyo Lee, Jeongduk Seo, Yong Hoon Shin, Pyung Chun Oh, Soon Yong Suh, Kyounghoon Lee, Woong Chol Kang, Taehoon Ahn, Seung Hwan Han

**Affiliations:** 1 Division of Cardiology, Department of Internal Medicine, Gachon University Gil Medical Center, Incheon 21565, Korea; albert.jang.md@gmail.com (A.Y.J.); mskimgene@gmail.com (M.K.); joonpyu@gilhospital.com (J.L.); jaidyseo@gilhospital.com (J.S.); fibrillary@gilhospital.com (Y.H.S.); likemed@gmail.com (P.C.O.); ssy@gilhospital.com (S.Y.S.); cardioman@gilhospital.com (K.L.); kangwch@gilhospital.com (W.C.K.); 2 Division of Cardiology, Department of Internal Medicine, Korea University Anam Hospital, Seoul 02841, Korea; ath3869@naver.com

**Keywords:** left main disease, percutaneous coronary intervention, coronary artery bypass surgery, long-term outcomes, real world, decision making

## Abstract

Background: The decision-making factors and long-term clinical outcomes between PCI and CABG in left main (LM) disease are still not well defined in the real world. Methods: We evaluated consecutive patients (*n* = 230) with LM disease either treated by PCI (*n* = 118) or CABG (*n* = 112). The primary endpoint was major adverse cardiovascular events (MACE), defined as a composite of cardiac death, spontaneous myocardial infarction (MI), stroke, and target vessel revascularization (TVR) for 7 years. Results: In the multivariate-adjusted analysis, the presence of intermediate EuroSCORE II and high SYNTAX scores predisposed to CABG. Isolated LM disease was associated with receiving PCI. The PCI group had a similar rate of MACE (HR_adj_ 0.97, 95% CI [0.48–1.94], *p* = 0.92) and a lower tendency of hard MACE (HR_adj_ 0.49, 95% CI [0.22–1.07], *p* = 0.07) compared to the CABG group, mainly due to the balance between a higher rate of TVR (HR_adj_ 9.71, *p* = 0.02) and a lower rate of stroke (HR_adj_ 0.22, *p* = 0.09) with the PCI group than in the CABG group. Conclusions: The decision making of treatment strategy was made based on clinical and angiographic factors. The selected patients who received PCI showed similar MACE and trend of a lower rate of composite hard endpoints despite multivariate adjustment.

## 1. Introduction

The optimal treatment strategy between a percutaneous coronary intervention (PCI) using drug-eluting stents (DES) or coronary artery bypass graft (CABG) surgery for left main (LM) coronary disease in real-world practice has been controversial. Previous randomized controlled trials (RCT) have shown that CABG was superior to PCI in terms of composite clinical endpoints, which included death, myocardial infarction (MI), stroke, and target lesion revascularization (TLR) [1,2,3,4]. However, this trend was only retained when the composites included TLR [1,2,3,4]. More importantly, mortality was similar among PCI and CABG in three out of the four major RCTs [1,2,3]. In one RCT trial, the Evaluation of XIENCE versus Coronary Artery Bypass Surgery for Effectiveness of Left Main Revascularization (EXCEL) reported higher mortality in the PCI group; this phenomenon was driven by non-cardiac deaths, which may have a weak etiologic relation with the initial revascularization strategy [4]. Moreover, the CABG-treated group had a higher rate of stroke in several trials [1,3,4]. Considering that some patients consider stroke as a deadly complication equivalent to death [5], a patient’s perception of optimal revascularization strategies may differ from those derived by composite outcomes in previous RCTs [6]. Due to these reasons, the optimal treatment strategy in patients with LM lesions remains debatable. Real-world evidence in such patients is crucial because RCT results may not necessarily translate to mundane practice [6,7]. In this study, we comparatively analyzed factors for selecting the treatment strategy and long-term clinical outcomes of real-world patients treated with PCI or CABG for LM lesions.

## 2. Materials and Methods

### 2.1. Study Design and Patient Population

**Figure 1:**
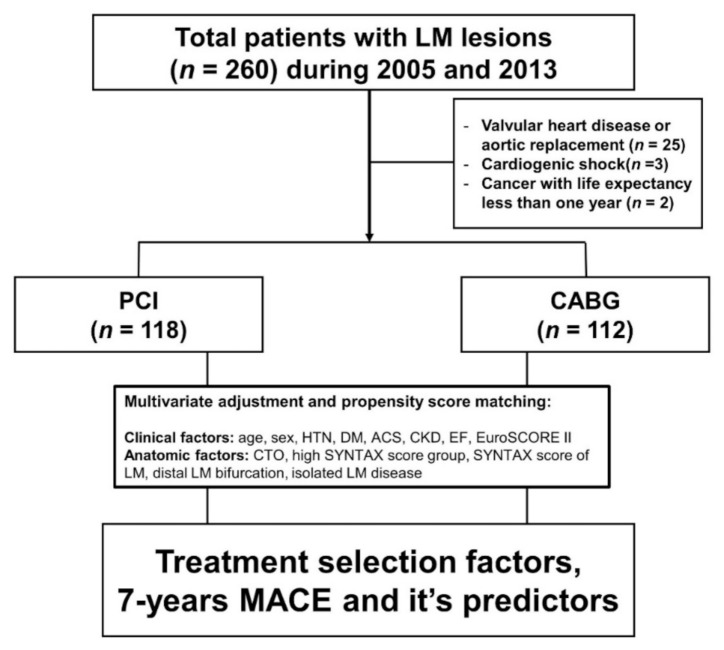
Study flow of enrolled patients. LM, left main; PCI, percutaneous coronary intervention; CABG, coronary artery bypass graft; HTN, hypertension; DM, diabetes mellitus; ACS, acute coronary syndrome; CKD, chronic kidney disease; EF, ejection fraction; CTO, chronic total obstruction; SYNTAX, SYNergy between percutaneous intervention with TAXus DES and cardiac surgery.

The current study was a single-center, retrospective, observational study of consecutive patients with LM disease undergoing either PCI (with drug-eluting stents) or CABG. To evaluate the long-term outcome, we analyzed patients receiving PCI or CABG between 1 August 2005 and 15 April 2013. The inclusion criteria were subjects with an LM disease treated by PCI or CABG who were ≥18 years of age. Those with (1) concurrent mitral or aortic valvular valve surgery, (2) congenital heart disease, (3) debilitating cancer with a life expectancy of less than one year, or (4) cardiogenic shock were excluded from the study. This study was approved by the institutional review boards of Gachon University Gil Medical Center (GDIRB2021-341, approved on 10 September 2021) and complied with the Declaration of Helsinki (6th revision). The study flow is shown in Figure 1.

### 2.2. Definition of Study Endpoints

The therapeutic strategy was assigned by attending doctors (cardiologists and cardiac surgeons) after discussing with patients and family members. We analyzed the independent factors in deciding either PCI or CABG. The primary endpoint was major adverse cardiovascular events (MACE), defined as a composite of cardiac death, spontaneous MI, stroke, and target vessel revascularization (TVR) for 7 years. Secondary endpoints included constituents of MACE and hard MACE. Hard MACE is a composite of hard endpoints, defined as cardiac death, spontaneous MI, and stroke. All clinical events were adjudicated by the consensus of two or more cardiologists. The cause of death was considered cardiac unless there was definite evidence of a non-cardiac cause. Spontaneous MI was defined as type 1, 2, and 3 MI based on the fourth universal MI definition [8]. TVR was defined as repeat revascularization by PCI or CABG in the previously treated vessel, when % diameter stenosis (DS) > 50% was associated with ischemic signs/symptoms or % DS > 70% with or without the presence of ischemic signs/symptoms.

### 2.3. Statistical Analysis

We analyzed data using IBM SPSS Statistics (IBM Corp. Released 2014. IBM SPSS Statistics for Windows, Version 23.0. Armonk, NY, USA: IBM Corp.) and R statistical software (version 3.6.0; R Foundation for Statistical Computing, Vienna, Austria). Continuous variables are presented as the mean ± SD for characteristics with appropriately near-symmetrical distributions or as median (interquartile range). Discrete data are presented as frequencies and percentages. Groups comparisons were evaluated with the Student’s *t*-test, the Mann–Whitney *U* test, or the Pearson χ^2^ test.

To evaluate each factor’s independent effect in deciding PCI or CABG and independent factors for long-term MACE, we performed a univariable and multivariable-adjusted binary logistic regression analysis after performing multivariate linear regression analysis to exclude variables with multicollinearity among adjusting variables.

For the 7-year clinical outcomes analysis, we used the Cox proportional hazards regression model. We included parameters either clinically relevant or statistically significant in the univariate analysis for subsequent multivariate analysis. Longitudinal data were plotted using the Kaplan–Meier estimates with log-rank tests and Cox proportional hazard model.

We additionally performed propensity score matching (PSM) to balance the baseline clinical and angiographic discrepancies between both groups. Propensity scores for each group were calculated using logistic regression analysis. Two groups were matched for 11 pre-procedural clinical and angiographic parameters (over 65 years of age, sex, hypertension, diabetes mellitus, chronic kidney disease [CKD], left ventricular ejection fraction [LVEF] over 40%, EuroSCORE II category, high SYNergy between percutaneous intervention with TAXus DES and cardiac surgery [SYNTAX] scores, SYNTAX score of LM, distal LM bifurcation, and isolated LM disease). Both groups were matched one-to-one with a caliper width of 0.2, using the nearest neighbor method.

## 3. Results

### 3.1. Baseline Characteristics

We enrolled a total of 260 consecutive patients with LM lesions. After excluding 30 patients (25 patients with concurrent valvular or aortic surgery, 3 patients presenting cardiogenic shock, and 2 patients with cancer who had less than one year of life expectancy) (Figure 1), a total of 230 patients were evaluated. The overall age was 64.1 ± 9.5 years, 72% were men, 59% had hypertension, and 35% had diabetes mellitus, while the mean LVEF was 58.8% (Table 1). There was no difference in demographic data between the PCI and CABG group. However, LVEF was significantly higher with PCI, while the EuroSCORE II was significantly higher in the CABG group (Table 1).

### 3.2. Angiographic and Procedural Characteristics

Table 2 demonstrates the baseline lesion characteristics. The SYNTAX score of the CABG group was significantly higher than the PCI group (Table 2). The SYNTAX score of LM lesions was also significantly more severe in the CABG group, although the PCI group was more frequent with low to intermediate SYNTAX scores (Table 2). The number of coronary lesions and the proportion of chronic total occlusive lesions (CTO) were also significantly higher in the CABG group. Isolated LM lesions and LM lesions with concurrent one additional vessel disease were more frequent in the PCI group. In contrast, LM lesion combined with two or three-vessel disease was more prevalent in the CABG group (Table 2).

Among patients who received PCI, the stent diameter and length in the main vessel were 3.4 and 20.9 mm, respectively. In the CABG group, the number of grafts used in each CABG surgery was 2.3. The internal mammary artery was used 94.6% of the time as a bypass vessel. The proportion of off-pump surgery was 42.9% (Table 2).

### 3.3. Factors for the Selection of PCI or CABG

**Figure 2:**
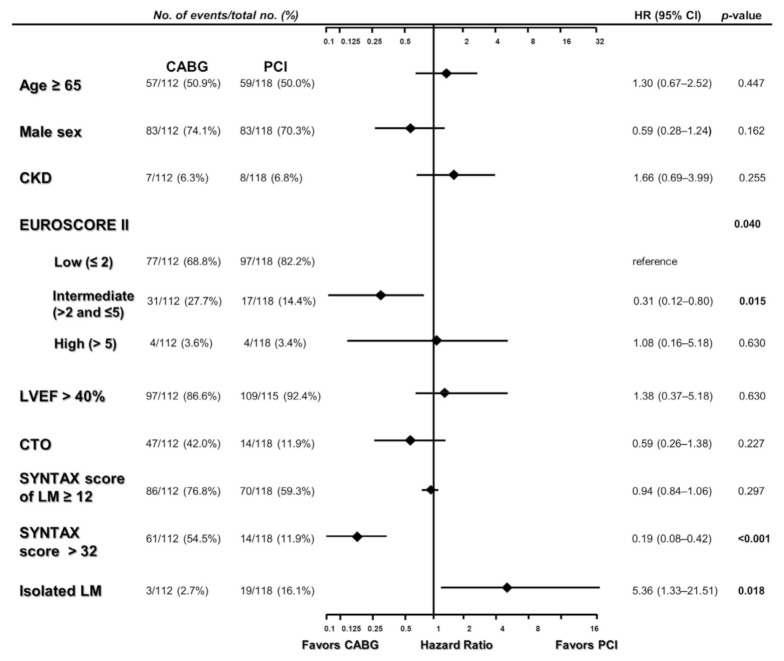
A multivariate forest plot of decision-making factors for PCI or CABG. Adjusted for age ≥ 65, sex, HTN, ACS, CKD, CTO, DM, EF, EuroSCORE II, HTN, presence of high SYNTAX scores group, SYNTAX score of LM, distal LM bifurcation, isolated LM disease. HR, hazard ratio; CI, confidence interval; CABG, coronary artery bypass graft; PCI, percutaneous coronary intervention; CKD, chronic kidney disease; LVEF, left ventricular ejection fraction; CTO, chronic total obstruction; SYNTAX, SYNergy between percutaneous intervention with TAXus DES and cardiac surgery; LM, left main. All other abbreviations including adjusted variables are listed in Table 1 and Table 2.

We then analyzed the determinants in either selecting PCI or CABG (Figure 2 and Supplemental Table S1). In the univariate analysis, intermediate risk of EuroSCORE II (>2 and ≤5), the presence of CTO, high SYNTAX score of LM (≥12), and high total SYNTAX score (>32) were favorable factors for the selection of CABG. On the other hand, preserved LVEF (>40%) and isolated LM disease were favorable factors for choosing PCI (Supplemental Table S1). In the multivariate analysis, intermediate risk of EuroSCORE II and high SYNTAX score (>32) were predictive of choosing CABG, whereas the presence of isolated LM disease predisposed to receiving PCI (Figure 2 and Supplemental Table S1).

### 3.4. Long Term Clinical Outcomes: Unadjusted Survival Analysis

**Figure 3:**
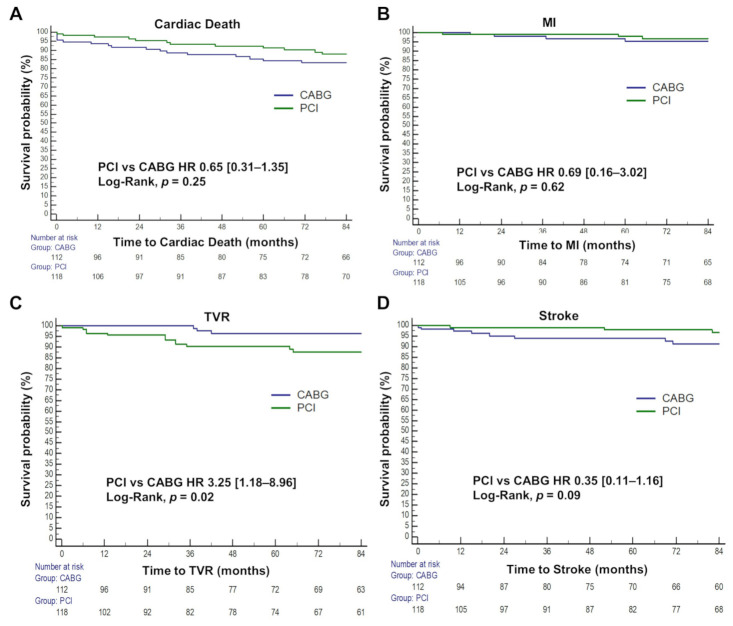
Kaplan–Meier survival analyses of clinical endpoints. Unadjusted Kaplan–Meier plots of (**A**) cardiac death, (**B**) myocardial infarction (MI), (**C**) stroke, and (**D**) target vessel revascularization (TVR) is shown. CABG, coronary artery bypass graft; PCI, percutaneous coronary intervention; HR, hazard ratio.

The median follow-up duration was 89 months (interquartile range, 32.0–127.5) (Supplemental Table S2). In the crude survival analysis, there was a significantly higher rate of TVR (HR 3.25, 95% CI 1.18–8.96, log-rank *p* = 0.02) and a trend of less stroke (HR 0.35, 95% CI [0.11–1.16], log-rank *p* = 0.09) with PCI. There was no difference in cardiac death and MI (Figure 3, Table 3, and Supplemental Table S2).

**Figure 4:**
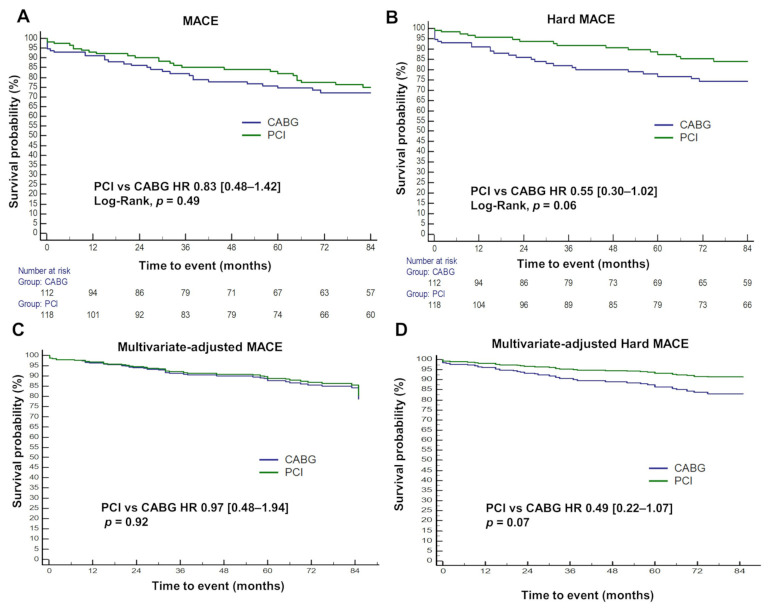
Unadjusted and multivariate-adjusted survival plot of composite endpoints. Kaplan-Meier plot of (**A**) major adverse cardiac events (MACE) and (**B**) hard MACE. The survival curve of multivariate-adjusted MACE and hard MACE are shown in (**C**,**D**), respectively. Adjusted for age ≥ 65, sex, HTN, ACS, CKD, CTO, DM, EF, EuroSCORE II, HTN, presence of high SYNTAX scores group, syntax score of LM, distal LM bifurcation, isolated LM disease. MACE, major adverse clinical events; CABG, coronary artery bypass graft; PCI, percutaneous coronary intervention; HR, hazard ratio. All other abbreviations including the adjusted variables are listed in Table 1 and Table 2.

MACE and hard MACE occurred in 53 (23.0%) and 42 (18.3%) individuals, respectively (Supplemental Table S2). The incidence of MACE was similar between two groups (PCI vs. CABG, HR 0.83, 95% CI [0.48–1.42], log-rank *p* = 0.49) due to a summation of contradicting dispositions of two constituent endpoints—TVR and stroke—neutralizing each other (Figure 4A, Table 3). On the other hand, CABG had a trend of a higher rate of hard MACE (HR 0.55, 95% CI [0.30–1.02], log-rank *p* = 0.06) (Figure 4B, Table 3), probably owing to the absence of TVR (Table 3).

### 3.5. Long Term Clinical Outcomes: Multivariate-Adjusted Survival Analysis and PSM

**Figure 5:**
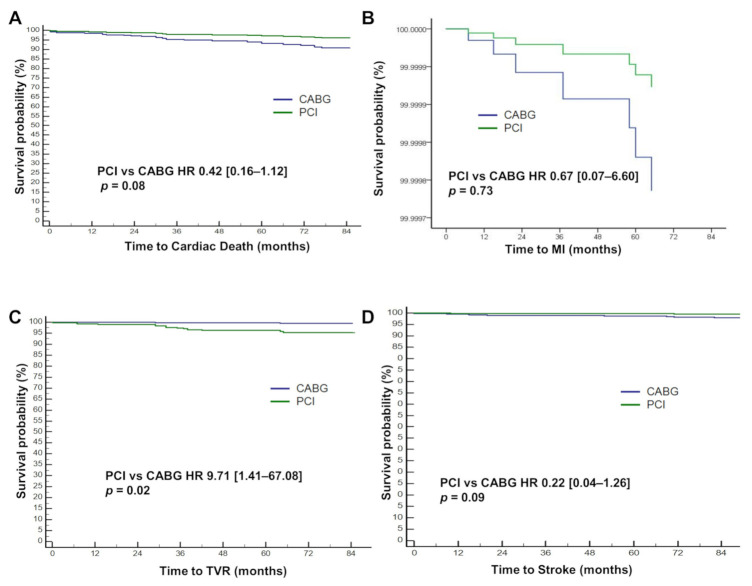
Multivariate-adjusted survival plot of clinical endpoints. Multivariate Cox regression plots of (**A**) cardiac death, (**B**) myocardial infarction (MI), (**C**) target vessel revascularization (TVR), and (**D**) stroke are shown. Adjusted for age ≥ 65, sex, HTN, ACS, CKD, CTO, DM, EF, EuroSCORE II, HTN, presence of high SYNTAX scores group, syntax score of LM, distal LM bifurcation, isolated LM disease. CABG, coronary artery bypass graft; PCI, percutaneous coronary intervention; HR, hazard ratio. All other abbreviations are listed in Table 1 and Table 2.

We further performed multivariate Cox regression to adjust for substantial baseline discrepancies. Results were consistent with the unadjusted analysis. PCI had a similar rate of MACE (HR 0.97, 95% CI [0.48–1.94], *p* = 0.92) compared with CABG (Figure 4C, Table 3). Cardiac death showed a trend of lower incidence (HR 0.42, 95% CI [0.16–1.12], *p* = 0.08) with PCI (Figure 5A, Table 3). Similar to the univariate analysis, TVR was significantly more frequent (HR 9.71, 95% CI [1.41–67.08], *p* = 0.02) while stroke was trending towards infrequent (HR 0.22, 95% CI [0.04–1.26], *p* = 0.09) in the PCI group (Figure 5C,D and Table 3). CABG was associated with a trend of harder MACE compared with PCI (HR 0.49, 95% CI [0.22–1.07], *p* = 0.07), consistent with the findings of the univariate Cox analysis (Figure 4D and Table 3).

We additionally performed PSM to balance for the baseline clinical and angiographic characteristics. After PSM, 66 patients were allocated to each group. There were no differences in baseline variables after matching (Supplemental Table S3). The post-PSM analysis of composite and secondary endpoints showed similar trends with the multivariate analysis. MACE was similar between groups (HR 1.01, 95% CI [0.49–2.10], *p* = 0.99), although PCI showed a trend for better prognosis in terms of hard MACE (HR 0.57, 95% CI [0.25–1.30], *p* = 0.18). The trends after PSM were consistent with the multivariate analysis; however, statistical significance was not achieved probably due to the reduced sample size (Table 3 and Supplemental Figures S1 and S2).

### 3.6. Predictors for Composite Outcomes

Table 4 shows independent predictors of the composite endpoints. In the multivariate analysis, HTN (HR_adj_, 2.53, 95%CI [1.11–5.78], *p* = 0.03), CKD (HR_adj_, 2.30, 95% CI [1.14–4.60], *p* = 0.02), and higher EuroSCORE II (HR_adj_, 1.54, 95% CI [1.23–1.93], *p* ≤ 0.01) were independent ominous predictors. Male sex (HR_adj_, 0.50, 95% CI [0.27–0.92], *p* = 0.03) was an independent favorable factor of MACE. CKD (HR_adj_, 2.30, 95% CI [1.06–4.95], *p* = 0.03) and higher EuroSCORE II (HR_adj_, 1.59, 95% CI [1.25–2.02], *p* ≤ 0.01) were independent unfavorable predictors of hard MACE. In addition, lesion characteristics (presence of CTO, SYNTAX score, location of LM disease) were not predictors for either of the composite endpoints (Table 4).

## 4. Discussion

In this study, the determinant factors for selecting the treatment strategy (PCI or CABG), long-term (7 years) clinical outcomes, and its predictors of real-world patients with LM diseases were analyzed. We found that (1) intermediate EuroSCORE II (2 < EuroSCORE II ≤ 5) and high SYNTAX scores were independent factors for assigning CABG; (2) PCI was selected significantly more in patients with isolated LM disease; (3) the incidence of MACE was comparable between two strategies, although this was ironically caused by the contradicting effects of constituent endpoints, TVR and stroke, canceling out each other after multivariate adjustment and PSM; (4) TVR was significantly more frequent with PCI, whereas cardiac death and stroke had a trend of higher incidence with CABG; (5) PCI had a trend of being associated with less hard MACE, a composite of hard endpoints, after multivariate adjustment and PSM.

To our knowledge, this is the first report to analyze the real-world decision-making factors of treatment strategy in patients with LM disease. Patients with intermediate risk of EuroSCORE II (2 < EuroSCORE II ≤ 5) were associated with a higher chance of receiving CABG compared with subjects with low EuroSCORE II (EuroSCORE < 2), demonstrating that clinical factors play a critical role in deciding the treatment strategy. Interestingly, however, when patients had a higher EuroSCORE (EuroSCORE > 5), CABG was no longer the preferred choice of treatment compared to those with low EuroSCORE II. Physicians and surgeons may prefer CABG with acceptable post-operative risk, as in the intermediate risk of EuroCORE II. However, PCI would paradoxically be preferred in subjects with higher postoperative risk (high EuroSCORE II) probably due to unsuitable coronary anatomy and relative contraindications to surgery [9].

We also demonstrated that lesion complexity is important in decision making. Disease extent such as SYNTAX score and the presence of isolated LM disease are important selection factors for PCI or CABG. As recommended in the guidelines, PCI was preferred in isolated LM disease as it has been previously shown to have comparable outcomes compared with CABG [10]. A high SYNTAX score was associated with deciding to perform CABG. It has previously been demonstrated that CABG is superior to PCI in subjects with high SYNTAX scores by randomized trials [11]. These results explain that cardiologists and cardiac surgeons understand that decision making of treatment strategy largely depends on the feasibility of treatment (clinical and angiographic complexity) also stressed by the treatment guidelines [10,12].

Interestingly, these decisions on treatment strategy by physicians and patients may translate to reduced hard endpoints and similar MACE in those receiving PCI compared with CABG in real-world practice. Most previous major RCTs showed that PCI was associated with significantly more composite endpoints mainly driven by TVR, although the rate of death was similar and the incidence of stroke was higher in the CABG group [1,2,3,4]. Consistent with such findings, the current study also showed that PCI was associated with more TVRs compared to CABG. This counterbalance between endpoints in the PCI group resulted in similar outcomes compared with CABG. Our results are meaningful because they show that patient selection for PCI by the responsible cardiologist results in similar or even better results compared with CABG in real-world practice despite multivariable adjustment or PSM [6,7]. These findings are also important because there are limited data regarding long-term real-word follow-up data for more than 5 years in those with LM disease.

PCI may also have undervalued advantages, which clinical trials may not be able to demonstrate. First, a higher risk of stroke after CABG surgery may be a huge obstacle for patients considering CABG, as many studies have shown. It has been demonstrated that patients would rather choose death over disabling stroke [5,13,14]. Our data show that after adjustment, the CABG group had a higher risk of having a stroke, consistent with previous trials [1,2,3,4]. These findings may be substantial evidence for patients choosing PCI, as stroke may be conceived as crucial as mortality, if not more so [5,13,14]. Moreover, repeat revascularization, TVR in our study, is conceived as an acceptable byproduct of PCI from the patient’s point of view [15]. Weighted endpoints [16], weighing stroke and death similarly, and TVR with less than half importance as shown by many surveys [15], may demonstrate that PCI is a reasonable option in LM disease, despite a higher chance of TVR.

### Limitations

Our data may have the intrinsic limitation of a single-center observational study. This indicates that results might be strongly influenced by local practices and may not be generalized to other patient groups. Additionally, the PSM analysis with an acceptable caliper width resulted in a much smaller sample size. Despite these difficulties, the PSM analysis showed similar trends with the findings from the multivariate-adjusted analysis. We also believe that factors such as the patient’s financial status or fear of surgery may have also played a role in determining PCI or CABG. Such unmeasurable factors may have confounded our analysis.

## 5. Conclusions

Decision making by the clinician and patient based on the clinical characteristics and lesion complexity to receive PCI in patients with LM diseases appear to be translated to similar or even better real-world long-term outcomes compared with CABG.

## Figures and Tables

**Table 1:** Baseline demographic and clinical characteristics.

	Total (*n* = 230)	PCI (*n* = 118)	CABG (*n* = 112)	*p*
Age (years)	64.1 ± 9.5	64.0 ± 10.3	64.28 ± 8.6	0.85
Male sex, *n* (%)	166 (72)	83 (75)	83 (69)	0.56
HTN, *n* (%)	136 (59)	66 (56)	70 (63)	0.35
DM, *n* (%)	81 (35)	37 (31)	44 (39)	0.22
Dyslipidemia, *n* (%)	53 (23)	31 (38)	22 (24)	0.27
Current smoker, *n* (%)	55 (24)	29 (25)	26 (20)	0.96
LVEF	58.8 ± 12.3	60.7 ± 11.1	56.8 ± 13.2	0.02
ACS, *n* (%)	155 (67)	74 (11)	79 (15.7)	0.26
PAD, *n* (%)	7 (3)	3 (3)	4 (4)	0.72
CKD, *n* (%)	15 (7)	8 (7)	7 (6)	1.00
Previous MI, *n* (%)	9 (4)	4 (3)	5 (5)	0.74
History of CVA, *n* (%)	14 (6)	8 (7)	6 (5)	0.79
History of PCI, *n* (%)	30 (13)	14 (12)	16 (14)	0.70
EuroSCORE II	1.65 ± 1.31	1.43 ± 1.35	1.90 ± 1.23	0.01

PCI, percutaneous coronary intervention; CABG, coronary artery bypass graft; HTN, hypertension; DM, diabetes mellitus; LVEF, left ventricular ejection fraction; ACS, acute coronary syndrome; PAD, peripheral arterial disease; CKD, chronic kidney disease; MI, myocardial infarction; CVA, cerebrovascular accident.

**Table 2:** Baseline lesions characteristics.

	Total (*n* = 230)	PCI (*n* = 118)	CABG (*n* = 112)	*p*
Lesions characteristics				
Total SYNTAX score	26.8 ± 11.2	20.7 ± 8.2	33.1 ± 10.4	<0.01
SYNTAX score of LM	12.8 ± 2.6	12.4 ± 2.2	13.1 ± 3.0	0.03
Low to intermediate SYNTAX score, *n* (%)	155 (67)	104 (88)	51 (46)	<0.01
Number of Lesions	3.3 ± 1.5	2.6 ± 1.3	4.0 ± 1.4	<0.01
CTO lesions, *n* (%)	62 (27)	15 (13)	47 (42)	<0.01
Distal LM bifurcation disease, *n* (%)	166 (72)	83 (70)	83 (74)	0.56
Extent of lesion				<0.01
Isolated LM disease, *n* (%)	21 (9)	18 (15)	3 (3)	
LM and 1-VD, *n* (%)	42 (18)	32 (27)	10 (9)	
LM and 2-VD, *n* (%)	59 (26)	37 (31)	42 (38)	
LM and 3-VD, *n* (%)	88 (38)	31 (26)	57 (51)	
Procedural characteristics				
Single-stent technique		93 (78.8)		
Two-stent technique		25 (21.2)		
Main vessel stent diameter (mm)	-	3.4 ± 0.4	-	-
Main vessel stent length (mm)	-	20.9 ± 8.5	-	-
Side branch stent diameter (mm)	-	3.1 ± 0.5	-	-
Side branch stent length (mm)	-	17.4 ± 4.0	-	-
Number of grafts	-		2.3 ± 0.5	-
Use of left internal mammary artery			106 (94.6)	
Off-pump surgery			48 (42.9)	

PCI, percutaneous coronary intervention; CABG, coronary artery bypass graft; SYNTAX, SYNergy between percutaneous intervention with TAXus DES and cardiac surgery; LM, left main; CTO, chronic total occlusion; VD, vessel disease.

**Table 3:** Unadjusted, multivariate adjusted, and post-PSM analysis of endpoints.

	PCI, *n* (%)	CABG, *n* (%)	HR (95% CI)	*p*
Cardiac death				
Unadjusted	12 (10.2)	17 (15.2)	0.65 (0.31–1.35)	0.25
Multivariable adjusted *	-	-	0.42 (0.16–1.12)	0.08
Post-PSM	7 (10.6)	11 (16.7)	0.63 (0.25–1.63)	0.34
MI				
Unadjusted	3 (2.5)	4 (3.6)	0.69 (0.16–3.02	0.62
Multivariable adjusted *	-	-	0.67 (0.07–6.60)	0.73
Post-PSM	3 (4.5)	3 (4.5)	0.66 (0.11–3.98)	0.65
TVR				
Unadjusted	12 (10.2)	3 (2.7)	3.25 (1.18–8.96)	0.02
Multivariable adjusted *	-	-	9.71 (1.41–67.08)	0.02
Post-PSM	7 (10.6)	1 (1.5)	7.33 (0.90–59.63)	0.06
Stroke				
Unadjusted	3 (2.5)	8 (7.1)	0.35 (0.11–1.16)	0.09
Multivariable adjusted *	-	-	0.22 (0.04–1.26)	0.09
Post-PSM	2 (3.0)	4 (6.1)	0.48 (0.09–2.60)	0.39
MACE				
Unadjusted	25 (21.2)	28 (25.0)	0.83 (0.48–1.42)	0.49
Multivariable adjusted *	-	-	0.97 (0.48–1.94)	0.92
Post-PSM	15 (22.7)	15 (22.7)	1.01 (0.49–2.10)	0.99
Hard MACE				
Unadjusted	16 (13.6)	26 (23.2)	0.55 (0.30–1.02)	0.06
Multivariable adjusted *	-	-	0.49 (0.22–1.07)	0.07
Post-PSM	9 (13.6)	15 (22.7)	0.57 (0.25–1.30)	0.18

* Adjusted for age ≥ 65, sex, HTN, ACS, CKD, CTO, DM, EF, EuroSCORE II, presence of high SYNTAX scores group, SYNTAX score of LM, distal LM bifurcation, and isolated LM disease. PSM, propensity score matching; MACE, major adverse clinical event; MI, myocardial infarction; TVR, target vessel revascularization; HR, hazard ration; CI, confidence interval. All other abbreviations including the adjusted variables are listed in Table 1 and Table 2.

**Table 4:** Predictors for the composite outcomes in LM disease.

Covariate		MACE			Hard MACE *	
	HR_adj_	95% CI	*p*	HR_adj_	95% CI	*p*
PCI vs. CABG	0.97	0.48–1.94	0.92	0.49	0.22–1.07	0.07
Age	0.99	0.95–1.04	0.73	1.03	0.98–1.08	0.30
Male	0.50	0.27–0.92	0.03	0.56	0.28–1.12	0.10
HTN	2.53	1.11–5.78	0.03	2.44	0.93–6.43	0.07
DM	0.91	0.50–1.67	0.77	0.99	0.50–1.96	0.97
ACS	0.60	0.30–1.18	0.14	0.60	0.26–1.36	0.22
CKD	2.30	1.14–4.60	0.02	2.30	1.06–4.95	0.03
LVEF	1.00	0.97–1.02	0.92	1.00	0.97–1.03	0.92
EuroSCORE II	1.54	1.23–1.93	<0.01	1.59	1.25–2.02	<0.01
CTO	1.52	0.69–3.35	0.30	1.33	0.54–3.30	0.54
SYNTAX score > 32	0.98	0.44–2.20	0.97	0.88	0.37–2.10	0.78
Distal LM bifurcation	1.43	0.69–2.96	0.34	1.09	0.49–2.45	0.83
Isolated LM disease	0.60	0.14–2.63	0.50	0.50	0.06–3.93	0.51

* Adjusted for age ≥ 65, sex, HTN, ACS, CKD, CTO, DM, EF, EuroSCORE II, HTN, presence of high SYNTAX scores group, syntax score of LM, distal LM bifurcation, isolated LM disease. MACE, major adverse cardiac event; HR_adj_, adjusted hazard ratio; CI, confidence interval; PCI, percutaneous coronary intervention. All other abbreviations including the adjusted variables are listed in Table 1 and Table 2.

## Data Availability

The data generated in this study are available from the corresponding author upon reasonable request.

## Supplementary Materials

The following supporting information can be downloaded at: https://www.mdpi.com/article/10.3390/jcm11030503/s1, Figure S1: Kaplan Meier plots of individual endpoints after propensity score matching; Figure S2: Kaplan Meier plots of composite endpoints after propensity score matching; Table S1: Decision-making factors for PCI strategy in LM disease; Table S2: Summary of clinical outcomes; Table S3: Baseline clinical and angiographic characteristics of pre- and post-propensity score matching.
