# Supplementary material for: Real-World Treatment Selection Factors and 7-Year Clinical Outcomes between Percutaneous Coronary Intervention and Coronary Artery Bypass Graft Surgery in Left Main Disease
Source: J Clin Med. 2022 Jan 19;11(3):503. doi: 10.3390/jcm11030503 (PMC8836954; doi:10.3390/jcm11030503)
Supplement: Supplementary file 1 [file jcm-11-00503-s001.zip › jcm-1484952-supplementary.pdf]

Journal of
Clinical Medicine

MDPI

Supplementary Materials: Real-World Treatment Selection Factors and 7-Year Clinical Outcomes between Percutaneous Coronary Intervention and Coronary Artery Bypass Graft Surgery in Left Main Disease

Albert Youngwoo Jang, Minsu Kim, Joonpyo Lee, Jeongduk Seo, Yong Hoon Shin, Pyung Chun Oh, Soon Yong Suh, Kyounghoon Lee, Woong Chol Kang, Taehoon Ahn and Seung Hwan Han

Table S1. Decision-making factors for PCI strategy in LM disease.

	Univariate Analysis			Multivariate Analysis *		
	**HR**	**95% CI**	***p***	**HR**	**95% CI**	***p***
Clinical variables						
Age ≥ 65	1.00	0.97–1.03	0.850	1.30	0.67–2.52	0.447
Male sex	0.83	0.47–1.48	0.520	0.59	0.28–1.24	0.162
DM	0.71	0.41–1.22	0.210	-	-	-
HTN	0.76	0.45–1.29	0.320	-	-	-
ACS	0.64	0.37–1.12	0.120	-	-	-
CKD	1.00	0.51–1.96	0.990	1.66	0.69–3.99	0.255
EUROSCORE II			0.048			0.040
Low risk (≤ 2)	ref	ref	ref	ref	ref	ref
Intermediate risk (> 2 and ≤5)	0.44	0.22–0.85	0.014	0.31	0.12–0.80	0.015
High risk (> 5)	0.79	0.19–3.28	0.750	1.08	0.16–7.24	0.939
LVEF > 40%	2.84	1.06–7.61	0.038	1.38	0.37–5.18	0.630
Lesion variables						
CTO	0.19	0.01–0.36	<0.001	0.59	0.26–1.38	0.227
SYNTAX score of LM ≥ 12	0.89	0.79–0.99	0.040	0.94	0.84–1.06	0.297
SYNTAX score > 32	0.11	0.06–0.22	<0.001	0.19	0.08–0.42	<0.001
Distal LM bifurcation	0.83	0.46–1.48	0.520	-	-	-
Isolated LM	6.97	2.00–24.28	<0.001	5.36	1.33–21.51	0.018

* Adjusted for age ≥ 65, sex, CKD, CTO, DM, EF, EuroSCORE II, LVEF, SYNTAX score, SYNTAX score of LM, and isolated LM disease. PCI, percutaneous coronary intervention; LM, left main; HR, hazard ratio; CI, confidence interval; DM, diabetes mellitus; HTN, hypertension; ACS, acute coronary syndrome; CKD, chronic kidney disease; LVEF, left ventricular ejection fraction; CTO, chronic total obstruction; SYNTAX, SYNergy between percutaneous intervention with TAXus DES and cardiac surgery. All other abbreviations including adjusted variables are listed in Table 1 and 2.

Table S2. Summary of clinical outcomes.

	**Total (*n* = 230)**	**PCI (*n* = 118)**	**CABG (*n* = 112)**	**Log-Rank-*p***
Median follow duration, months (IQR)	89 (32.0–127.5)	94 (53.6–128.0)	87.5 (27.5–128.3)	*0.60*
MACE, *n* (%)	53 (23.0)	25 (21.2)	28(25)	0.49
Hard MACE, *n* (%)	42 (18.3)	16 (13.6)	26 (23.2)	0.06
Cardiac death, *n* (%)	29 (12.6)	12 (10.2)	17 (15.2)	0.25
Spontaneous MI, *n* (%)	7 (3.0)	3 (2.5)	4 (3.6)	0.62
Stroke, *n* (%)	11 (4.8)	3 (2.5)	8 (7.1)	0.09

TVR, *n* (%)	15 (6.5)	12 (10.2)	3 (2.7)	0.02

Data are expressed number (%) or IQR, interquartile range. PCI, percutaneous coronary intervention; CABG, coronary artery bypass graft; IQR, interquartile range; MACE, major adverse cardiovascular events; MI, myocardial infarction; TVR, target vessel revascularization.

Table S3. Baseline clinical and angiographic characteristics of pre- and post-propensity score matching.

	Pre-PSM			Post-PSM		
	**PCI (*n* = 118)**	**CABG (*n* = 112)**	***p***	**PCI (*n* = 66)**	**CABG (*n* = 66)**	***p***
Age (years)	64.0 ± 10.3	64.3 ± 8.6	0.85	64.1 ± 8.8	66.0 ± 9.8	0.24
Male sex, *n* (%)	83 (75)	83 (69)	0.56	46 (69.7)	39 (59.1)	0.28
HTN, *n* (%)	66 (56)	70 (63)	0.35	38 (57.6)	38 (57.6)	1.00
DM, *n* (%)	37 (31)	44 (39)	0.22	25 (37.9)	26 (39.4)	1.00
Dyslipidemia, *n* (%)	31 (38)	22 (24)	0.27	15 (22.7)	12 (18.2)	0.67
LVEF	60.7 ± 11.1	56.8 ±13.2	0.02	59.1 ± 12.2	60.0 ± 11.8	0.68
ACS, *n* (%)	74 (11)	79 (15.7)	0.26	45 (68.2)	36 (54.5)	0.15
CKD, *n* (%)	8 (7)	7 (6)	1.00	4 (6.1)	9 (13.6)	0.24
EuroSCORE II	1.4 ± 1.4	1.9 ± 1.2	0.01	1.7 ± 1.0	1.7 ± 1.7	0.98
Low to intermediate SYNTAX score, *n* (%)	104 (88)	51 (46)	<0.01	50 (75.8)	50 (75.8)	1.00
Number of Lesions	2.6 ± 1.3	4.0 ± 1.4	<0.01	4.2 ± 5.4	4.5 ± 6.0	0.74
CTO lesions, *n* (%)	15 (13)	47 (42)	<0.01	19 (28.8)	11 (16.7)	0.15
Distal LM bifurcation disease, *n* (%)	83 (70)	83 (74)	0.56	47 (71.2)	44 (66.7)	0.71

PSM, propensity score matching; PCI, percutaneous coronary intervention; CABG, coronary artery bypass graft; MACE, major adverse clinical event; MI, myocardial infarction; TVR, target vessel revascularization. All abbreviations are listed in Table 1 and 2.

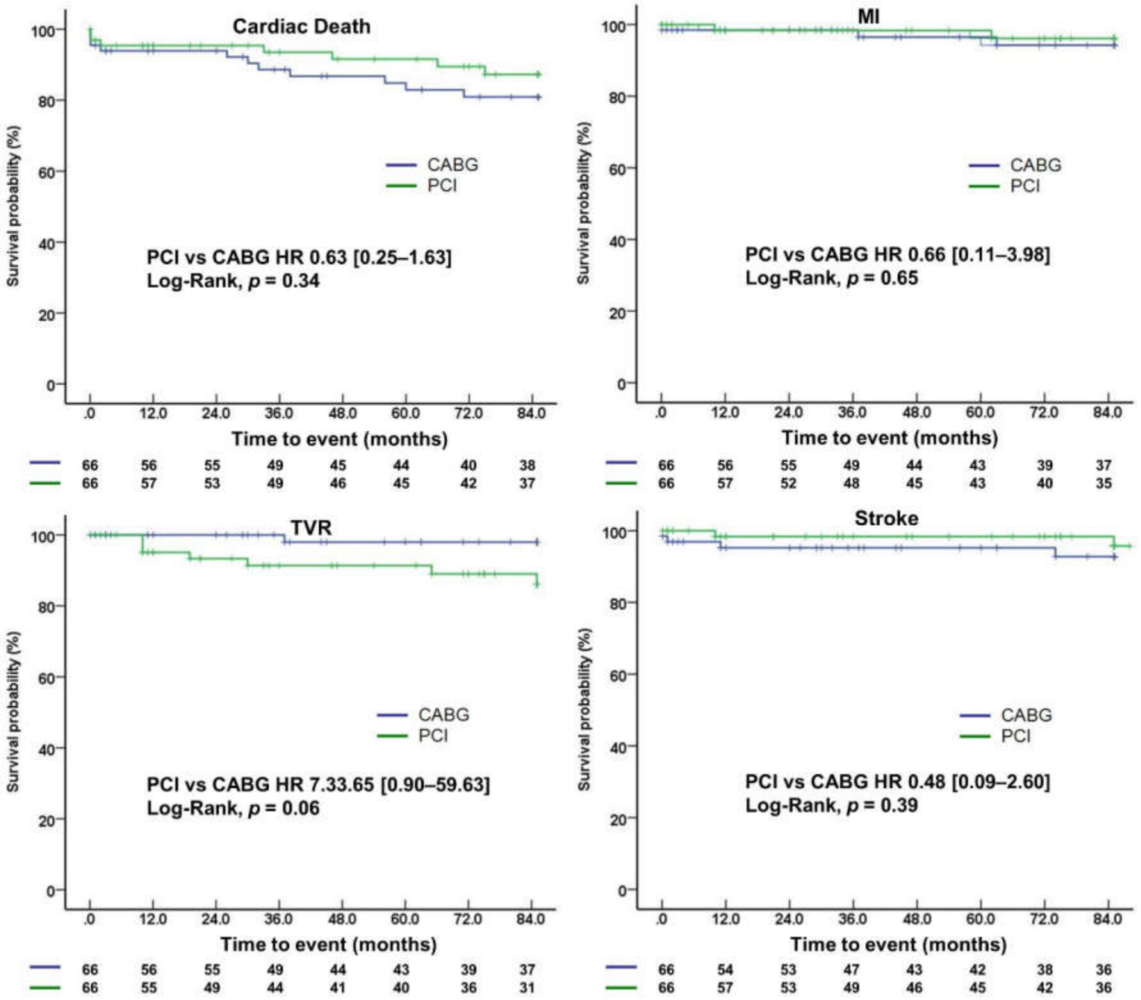

Figure S1. Kaplan Meier plots of individual endpoints after propensity score matching. All abbreviations are listed in Figure 3.

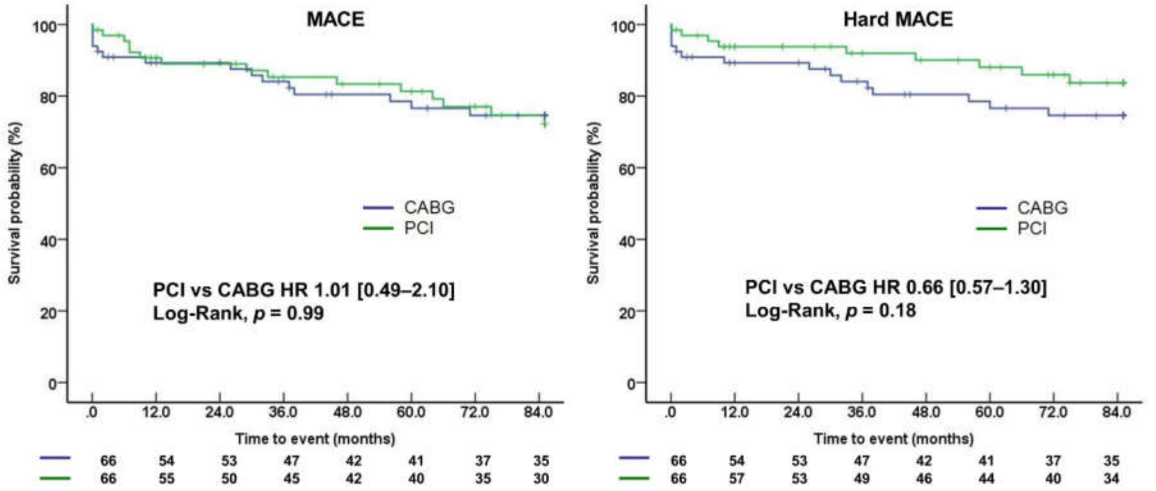

Figure S2. Kaplan Meier plots of composite endpoints after propensity score matching. All abbreviations are listed in Figures 3 and 4.
